# The significance of tubular and glomerular proteinuria in critically ill patients with severe acute kidney injury

**DOI:** 10.12669/pjms.306.5684

**Published:** 2014

**Authors:** Christopher Thiam Seong Lim, Han Khim Tan, Yeow Kok Lau

**Affiliations:** 1 Christopher Thiam Seong Lim, Department of Medicine, Faculty of Medicine and Health Sciences, University Putra Malaysia, Serdang, Malaysia.; 2 Han Khim Tan, Department of Renal Medicine, Singapore General Hospital, Singapore.; 3 Yeow Kok Lau, Department of Renal Medicine, Singapore General Hospital, Singapore.

**Keywords:** Acute kidney injury, Dialysis, Electrophoresis, Proteinuria, APACHE

## Abstract

***Objective:*** Critically ill patients with acute kidney injury (AKI) frequently need acute renal replacement therapy (aRRT). We evaluated an inexpensive, rapid quantitative and qualitative analysis of proteinuria on the course of AKI patients requiring aRRT in intensive care.

***Method: ***This was a prospective, observational study of critically ill patients with severe established AKI or Acute on Chronic Kidney Injury (AoCKI) requiring aRRT. Urine samples were analyzed using Sodium-Dodecyl-Sulphate-Polyacryamide Gel Electrophoresis (SDS-PAGE).

***Results:*** A total of 30 critically ill patients were studied. Those who died have higher APACHE II (29 ± 6 vs. 20 ± 5, p<0.001), multi-organ failure (0.7 ± 0.5 vs. 0.2 ± 0.4, p < 0.02) and Tubular/Glomerular ratio (114 ± 60 vs. 75± 37, p < 0.05).The renal non-recoverers have higher baseline creatinine (415 ± 328 vs. 125± 19 umol/l, p < 0.01), urinary Dipstick value (1.8±0.8 vs. 0.5±0, p <0.05) and Glomerular score (3.0 ± 1.8 vs. 0.6 ± 0.2, p < 0.02).Heavy tubular proteinuria also predicts a longer duration of interim dialysis support and mortality whereas glomerular proteinuria correlates with development of chronicity and End Stage Renal Disease (ESRD).

***Conclusions: ***The dominant presence of tubular proteinuria is associated with poor survival in patients who have high APACHE II score and multi-organ failure. It also correlates with a longer duration of dialysis support in survivals. Renal Non-recoverers had heavy dominant presence of glomerular proteinuria. SDS-PAGE proteinuria analysis offers a reliable and inexpensive method to prognosticate proteinuria in this group of critically ill patients.

## INTRODUCTION

Acute kidney injury (AKI) or acute-on-chronic kidney injury (AoCKI) are common occurrences amongst patients admitted to intensive care units (ICUs) with incidence ranges from 20 – 50%.^1^ The predominant cause of AKI or AoCKI is acute tubular necrosis (ATN). Beside high mortality rate, ATN is associated with adverse short and long term outcomes.^2^Traditional blood marker such as serum creatinine measurement has poor sensitivity and specificity because patients with AKI are not in steady state.^3^

Lately, there has been interest in using proteinuria as a more direct biomarker of kidney injury. A large series of clinical trials found a significant correlation between the extent of proteinruia and progression to ESRD.^4^ However, the association of proteinuria with AKI or AoCKI and its long term implication is less certain.

 The discovery of specific urinary tubular proteins, Cystatin-C, Neutrophil Gelatinase-Associated Lipocalin (NGAL) and Kidney Injury Molecule-1 (KIM-1) as indicators of tubular damage has received much attention.^5^ The appearance of these biomarkers are associated with unfavorable outcome as reflected by the requirement for aRRT.^6^ Although these tests are promising, they are not routinely available and have their own limitations.

On the other hand, semi-quantitative analysis of proteinuria by using SDS-PAGE offers inexpensive, rapid and convenient method of assessing the level (glomerular versus tubular) and extend of renal impairment.^7^ SDS-PAGE separates urinary proteins by their molecular sizes. The staining intensity of protein bands indicate the amount of protein loss in the urine, the presence of low molecular weight (LMW) protein bands reveal tubular protein loss, whereas the presence of albumin and high molecular weight (HMW) protein bands are evidence of glomerular damage. With the dedicated instrument (Phastsystem, Pharmacia, Sweden), precast gel and buffer strips, the test can be completed in less than two hours. Apart from the capital investment of S$17,000 (USD 10,000) on the instrument, the material cost for each test is only S$3.5(USD2.5). It is cheap enough to be made available as a routine test.

The aim of our study was to evaluate whether the results of tubular and glomerular proteinuria quantification by SDS-PAGE would yield any useful clinical information in term of patient and renal survival in critically ill patients with AKI or AoCKI.

## METHODS

This was a prospective, observational, single-center study. Severe AKI and AoCKI that are unresponsive to medical treatment in the intensive care units (ICUs) are referred to the renal department that will provide aRRT as clinically indicated. In this study, aRRT includes intermittent hemodialysis, slow efficiency extended dialysis and continuous venovenous hemofiltration. Choice of specific aRRT modality was determined by the patient’s hemodynamic, metabolic and nutritional status. We recruited 30 consecutive patients from ICUs who were referred to us for nephrology consultation who subsequently needed aRRT. All the patients recruited were in the RIFLE criteria, class “Failure” staging.

Fresh urine samples via indwelling urinary catheters were obtained during the first day of renal consultation. The samples were centrifuged and stored at –30ºC before subsequent batch analysis using SDS-PAGE technique on a PhastSystem (Pharmacia, Sweden) using PhastGel Gradient 8-25 and PhastGel SDS Buffer strips according to manufacturer’s instructions.^7^

**Fig.1:**
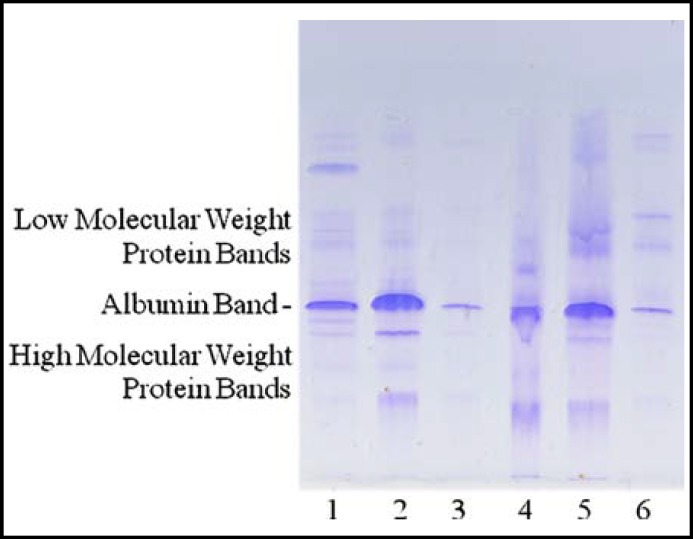
Separation of urinary protein by SDS-PAGE. Visual scoring on a scale of 0, 0.5, 1, 2, 3 & 4. Glomerular score (G): base on intensity of Albumin plus High Molecular Weight Protein Bands. Tubular score (T): base on intensity of Low Molecular Weight Protein Bands. Lanes: (1) G2T3, (2) G4T2, (3) G0.5T0.5, (4) G2T2, (5) G4T4, (6) G0.5T2.

The intensity and pattern of the staining is expressed in Glomerular Score and Tubular Score. Visual scoring by a single trained lab scientist was done on a scale of 0, 0.5, 1, 2, 3 & 4 and multiplied by dilution factor where applicable. LMW protein bands above Albumin (Fig.1) suggest presence of tubular injury and were assessed for the Tubular Score. Albumin plus the high molecular weight protein bands (HMW) below (Fig.1) suggest presence of glomerular damage and were assessed for the Glomerular Score.

This study was non-interventional and did not influence the nature of treatment received by the subjects. Institutional review board (IRB) approval was obtained and either the patients or patients’ closest available next-of-kin were approached in all cases for informed consent using IRB-approved informed consent form. This was an investigator-initiated study.

For the purpose of the study, patient survival is defined as patients that are alive at the time of discharged. Renal recovery is defined as renal function that has returned to normal or baseline value at the time of discharged. Renal non-recovery is defined as renal function that has deteriorated to either Impaired Renal Function (IRF) or End Stage Renal Disease (ESRD defined as eGFR <15 mL/min per 1·73 m2; chronic dialysis). Normal renal function (NRF) was defined as serum creatinine (SeCr) =< 140 umol/L.

***Statistical analysis:*** Summary descriptive data are reported as mean SD. Categorical data are presented as absolute numbers with percentages in parenthesis. Comparative univariate analysis for continuous data was performed using the Student t-test and categorical data by Pearson Chi-Square test. The proprietary statistical software package SPSS Version 20.0 for Windows (SPSS Inc, Chicago, IL, USA), was used for all statistical analyses.

## RESULTS

A total of 30 patients (mean age 58.5 ± 27.5 years; M:F=18 : 12) with severe renal injury (AKI : AoCKI 17 : 13) were prospectively studied for 3 months duration from Sep 2008 – Dec 2008.. All the patients were critically ill and had severe AKI or AoCKI that was resistant to standard medical therapy. All of them were subsequently initiated on aRRT in ICU.

As per other studies, the overall mortality was 11 out of 30 (37%).^1^ The non-survivors were significantly more ill at the time of the referral (APACHE II score: 29±6 vs. 20±5, p=0.001) (Table-I). They have lower baseline creatinine (122 ± 46 vs. 334 ± 307 umol/l, p <0.02) but suffer from higher numbers of multi-organ failure (0.7 ± 0.5 vs. 0.2 ± 0.4, p, 0.02). They also have higher tubular/glomerular ratio (114 ± 60 vs. 75± 37, p < 0.05) which indicates severe ATN.

Among the 19 survivors (63%), 5 patients (17%) were renal recoverers who achieved normal renal function (NRF) whereas 14 patients (46%) were non-recoverers with irreversible deterioration of renal function that progressed to either IRF or ESRD (Table-II). Compared renal recoverers with non-recoverers, both groups had similarly low Apache 2 scores and multi-organ failure scores at presentation. Besides a higher value of baseline serum creatinine, what distinguished renal non-recoverers from recoverers were higher glomerular score and tubular + glomerular total score indicating severe and irreversible insults to the glomeruli.

After excluding for survivors who developed ESRD, there were 6 patients who required =< 2 days and 8 patients who required >2 days of interim dialysis before full renal recovery. The group who required > 2 days of interim dialysis has significantly higher Tubular score (2.0 ± 1.1 vs. 0.7 ± 0.3, p < 0.02), and Glom +Tub Score (5.0 ± 2.8 vs. 1.7±1.2, p<0.02), suggesting that the tubular injury is reversible if the patients survived.

## DISCUSSION

Over the last decade, there have been many promising biomarkers such as Cystatin-C, NGAL, KIM-1 that assess the acute renal dysfunction.^8^ The availability of these new markers offers an unprecedented opportunity for improved evaluation and management of patients with AKI. To a larger extend, the current evidence of using them routinely has been limited. The use of the new biomarkers are also confounded by the fact that majority of the studies used serum creatinine as gold standard for validation, thereby limiting the clinical utility to establishing an earlier diagnosis of AKI.^8^ The combination of biomarkers together with proteinuria may further improve the accuracy of prediction of risk of developing AKI.^9^

**Table-I:** Summary demographic, clinical and laboratory data for Survivor vs. Non-survivor

	**Survivor**	**Non-survivor**	**p value**
Numbers (%)	19(63%)	11(37%)	-
APACHE II score	20±5	29±6	<0.001
Age (years)	65±17	64±8	NS
Baseline creatinine (umol/L)	334±307	122±46	<0.02
Intradialytic creatinine (umol/l)	402±209	353±128	NS
Multiorgan failure (yes=1, no=0)	0.2±0.4	0.7±0.5	<0.02
Dipstick score (0, 0.5, 1, 2, 3 & 4)	1.4 +/- 0.9	1.3 +/- 0.8	NS
Glomerular score (0, 0.5, 1, 2, 3 & 4)	2.3±1.9	1.5±1.0	NS
Tubular score (0, 0.5, 1, 2, 3 & 4)	1.4±1.4	1.5±1.0	NS
Tub/Glom ratio	75 ± 37	114 ± 60	<0.05
Tub + Glom total score	3.8 ± 2.5	3.1 ± 1.9	NS

* *Summary data expressed as mean* *SD* *(SD).* *A value of p < 0.05 was considered statistically significant. *

**Table-II:** Summary demographic, clinical and laboratory data for Renal recoverer vs. Non-recoverer

	**Recoverer**	**Non-recoverer**	**p value**
Numbers (%)	14(47%)	5(17%)	-
APACHE II score	19±5	21±5	NS
Age (years)	59±7	66±8	NS
Baseline creatinine (umol/L)	125±19	415±328	<0.01
Intradialytic creatinine (umol/l)	332±88	428±238	NS
Multiorgan failure	0.2±0.4	0.3±0.5	NS
Dipstick	0.5 +/- 0.0	1.8 ±0.8	<0.001
Glomerular score	0.6±0.2	3.0 ±1.8	<0.002
Tubular score	0.6±0.2	1.7 ±0.9	<0.02
Tub/Glom score	100 ± 0.0	66 ±39	<0.01
Tub + Glom score	1.2 ± 0.4	4.7±2.3	<0.005

* *Summary data expressed as* *mean SD.* *A value of p < 0.05 was considered statistically significant. *

**Table-III:** Comparing the duration of interim dialysis (Exclude those who died or became ESRD).

**Days Dialyzed**	**=<2**	**> 2**	**p value**
Numbers	6	8	-
Day Dialyzed	1.5 ± 0.5	9.9 ± 8.5	<0.05
Apache II score	19 ± 5	23 ± 5	NS
Baseline creatinine (umol/L)	158 ± 81	163 ± 75	NS
Multiorgan failure	0.2 ± 0.4	0.5 ± 0.5	NS
Sex, M:F	5 : 1	4 : 4	NS
Age, years	63 ±10	65 ± 9	NS
Dipstick	0.8 ± 0.6	1.7 ±1.0	=0.059
Glomerular score	1.0 ± 1.0	3.1 ± 2.2	=0.059
Tubular score	0.7 ± 0.3	2.0 ± 1.1	<0.02
Tub/Glom score	89 ± 27	73 ± 43	NS
Tub + Glom score	1.7 ± 1.2	5.0 ± 2.8	<0.02

* *Summary data expressed as* *mean SD.* *A value of p < 0.05 was considered statistically significant. *

During ATN there is an increase in the urinary excretion of an array of urinary proteins and enzymes.^10^ The proximal tubules and medullary thick ascending limb are particular susceptible to the ischaemic injury when AKI occurs. This is attributed by their high tubular energy requirement and also the paucity of blood supply.

Tubular proteinuria is not readily detected by routine dipstick examination. SDS-Page method is one of the few and unique methods to get hold of the tubular proteinuria rapidly at an affordable cost.^7^

Tubular proteinuria essentially consists of LMW proteins such as beta2-microglobulin, NGAL, KIM-1, Cystatin C and alpha1-microglobulin. They are filtered across the glomerular and are essentially reabsorbed by the proximal tubular cells under normal healthy condition. But interference with tubular function, due to a variety of tubulointerstitial diseases, can lead to appearance of tubular proteins.^11^

In critically ill patients with AKI, increased urinary tubular proteinuria can identify patients who required aRRT.^12^ In our study, we found that those who needed a longer duration of interim dialysis support have heavier tubular proteinuria. The appearance of tubular proteinuria may precede glomerular proteinuria in critically ill patient with AKI.^6^ Thus tubular proteinuria appeared to be a more reliable marker than glomerular proteinuria in predicting the length of interim support for critically patients who has AKI.^13^

We also found the presence of tubular proteinuria correlate with increased mortality.^1^ One possible explanation is that more critically ill patients have more extensive and severe ATN. The lower baseline creatinine for non-survivors implied that they were previously healthy individuals with no prior renal impairment. They unfortunately succumbed to death rapidly because of the high numbers of the multi-organ failure.

The appearance of middle and high molecular weight proteins, such as albuminuria has long been associated with increased mortality.^14^ However its value in predicting AKI in ICU setting has never been reported. Although urinary dipstick has good specificity for proteinuria, it is not as sensitive as quantitative methods such as SDS-PAGE.

In our study, the renal non-recoverers were found to have significantly higher baseline serum creatinine and higher glomerular proteinuria scores. We demonstrated that their presence in urine is associated with poor renal survival. This concurred with the cohort study done by Matthew where his team has shown that progressive renal injury is significantly increased with the presence of glomerular proteinuria.^15^

A limitation of this study is the small number of patients investigated. The ability to predict mortality accurately would require study of a much larger and heterogenous cohort than our present study.

In summary, SDS-PAGE analysis is shown to be a cheap and reliable method in defining the presence of tubular and glomerular proteinurias in severe AKI patients requiring aRRT in ICUs. The presence and intensity of tubular proteinuria correlate the duration of dialysis support and mortality, whereas the appearance and intensity of glomerular proteinuria indicate severe and irreversible glomerular damage. This valuable laboratory information could aid the physician in day-to-day management of AKI cases in the ICUs.
